# Consumer-oriented review of digital diabetes prevention programs: insights from the CDC’s diabetes prevention recognition program

**DOI:** 10.3389/fcdhc.2025.1562108

**Published:** 2025-04-16

**Authors:** Benjamin Lalani, Jalene Shim, Vidhu Vadini, Yllka Valdez, Daniel Zade, Nestoras Mathioudakis

**Affiliations:** Division of Endocrinology, Diabetes & Metabolism Johns Hopkins University School of Medicine, Baltimore, MD, United States

**Keywords:** prediabetes, diabetes, lifestyle intervention, digital health, diabetes prevention, cdc, mHealth, artificial intelligence

## Abstract

**Background:**

Prediabetes is highly prevalent and significantly increases the risk of type 2 diabetes. While access to proven interventions like the Diabetes Prevention Program (DPP) has historically been limited, digital DPPs (dDPPs) present a promising and scalable option. With the recent growth of dDPP offerings and potential variability across platforms, access to accurate and clear information is crucial for individuals seeking diabetes prevention options. This review provides an overview of the dDPP landscape and characterizes the “direct-to-consumer” information available–or lacking–for patients choosing a dDPP.

**Methods:**

We identified dDPPs through the CDC Diabetes Prevention Recognition Program (DPRP) Registry. Data were extracted from three sources available to consumers: the CDC DPRP Registry, the CDC “Find a Lifestyle Program” Website, and program-specific websites. Extracted data included CDC recognition status, intended audience, available languages, program features (e.g., artificial intelligence, integration with smart devices), website availability and functionality, demonstrations of credibility (e.g., ADA endorsement), clinical performance metrics (e.g., average weight loss), and user experience factors (e.g., satisfaction). Descriptive statistics were used to summarize extracted data.

**Results:**

A total of 97 dDPPs were included in the review, with most in the early stages of CDC recognition. Only 35% of dDPPs listed in the CDC registry had functional websites, though additional websites were identified through manual searches. Program-specific features included AI-driven health recommendations, device integration (e.g., digital scales and activity trackers), nutrition tracking tools, and telehealth platforms. Nearly half of the dDPPs reported clinical performance metrics such as weight loss and A1C outcomes. User experience details were often presented through patient testimonials and satisfaction scores. Notably, many programs required users to provide personal information to access additional information.

**Conclusion:**

We found that available dDPPs vary significantly in their features, designs, and structures, reflecting a diverse and evolving landscape of diabetes prevention options. Concurrently, many dDPPs lack accessible information due to missing or incomplete websites. Centralized sources of information provided by the CDC are also insufficient, with discrepancies and gaps that hinder transparency and consumer decision-making. Addressing these issues through enhanced program visibility and improved centralized databases will be critical to optimizing the reach and impact of dDPPs.

## 1 Introduction

Prediabetes is a high-risk state for type 2 diabetes, characterized by elevated glucose or A1C levels that exceed normoglycemia but do not meet the threshold for diabetes (1). It is a major public health concern affecting an estimated 97.6 million U.S. adults (2). Globally, the prevalence of prediabetes exceeds 9%, with projections indicating that nearly 1 billion people could be affected by 2045 (3). Without intervention, approximately 10% of individuals with prediabetes progress to type 2 diabetes annually (4). Therefore, this condition represents a crucial window for prevention efforts to mitigate the substantial morbidity and healthcare costs associated with diabetes and its complications.

One effective intervention is the Diabetes Prevention Program (DPP), which offers structured lifestyle and behavior modification education focusing on nutrition and physical activity (5). The landmark DPP Trial demonstrated that these interventions reduce the risk of type 2 diabetes by 58% over three years, outperforming metformin (6). Long-term follow-up from the Diabetes Prevention Program Outcomes Study (DPPOS) found that the risk reduction persisted at 27% even 15 years after program completion (7). However, despite the proven benefits, access to DPPs remains a significant barrier, with only one DPP center for every 45,000 adults with prediabetes (8). Rural areas face even greater disparities, with only 15% of counties offering DPPs compared to 50% in urban areas (9).

To address these access issues, the DPP was adapted into a digital format. In 2015, the National DPP introduced an online option, allowing remote participation via online platforms and mobile apps (10). There is evidence that digital Diabetes Prevention Programs (dDPPs) may be as effective as traditional in-person programs (11), with some research even suggesting that digital formats may offer advantages in terms of retention and participation rates (12).

The Center for Disease Control and Prevention (CDC) Diabetes Prevention Recognition Program (DPRP) certifies digital DPPs, ensuring they adhere to an approved curriculum, offer coaching interactions, and achieve key outcomes such as participant engagement, physical activity tracking, and weight loss. Since the introduction of digital options in 2015, the number of enrollees in dDPPs has grown nearly fourfold in just four years (10). The number of recognized digital programs has also expanded from just 4 in 2015 to 35 in 2019 (10); today, there are 97 programs and counting.

With the growing variety of dDPPs available, consumers now have more choices when selecting a program that meets their needs. The study aimed to assess the quality and clarity of information available to individuals with prediabetes seeking a digital DPP. Our analysis used information available from the CDC DPRP Registry, the CDC “Find a Lifestyle Change Program” website, and program-specific websites (13, 14). We evaluated key factors, including delivery platforms (website, app, or both), program credibility (e.g., CDC recognition, peer-reviewed publications, certifications), and reported clinical outcomes (e.g., weight loss and physical activity improvements). Additionally, we reported on the use of unique features such as AI integration, smart device compatibility, and user experience elements like eligibility requirements and access barriers. This review aims to provide insights into how effectively the CDC and CDC-recognized dDPPs present information to support informed decision-making for individuals pursuing diabetes prevention and management options.

## 2 Methods

### 2.1 Identification of dDPPs

The dDPPs included in this review were identified using the CDC’s DPRP Registry, accessed on November 8, 2024 (13). From the CDC-recognized DPPs, we included only those designated as “online non-live.” Each program’s associated website was reviewed (if it was available) to assess the availability of program-specific information. A subset of dDPPs was selected for a deep-dive analysis if their associated website provided sufficient information for characterization. This was defined by the following criteria: a dedicated website specific to their dDPP, detailed information on program features and performance (rather than generic information about the national DPP), and clear evidence that the program operated as a digital, non-live dDPP. Programs lacking such information or clarity about their digital, non-live format were excluded from the deep-dive analysis.

### 2.2 CDC DPRP registry and find a program locator data extraction and validation

We utilized two CDC-based sources of information: the DPRP Registry (13) and the “Find a Program” Locator (14), both of which provided unique details about the CDC-recognized programs. Data from these sources were extracted and compiled using REDCap, a secure electronic data capture tool hosted at Johns Hopkins University. The data extraction process was conducted between November 8, 2024, to November 31, 2024. The REDCap data collection instruments are provided in the Supplemental Document.

From the CDC DPRP Registry, we extracted data on program name, address, website, recognition status (Pending, Preliminary, Full, or Full Plus), and intended audiences. From the Find a Program Locator, we recorded the listed website, information on program features (e.g., mobile app compatibility, provision of digital scales, telehealth options), language available (e.g., English, Spanish), and payment structures (e.g., free of charge, self-pay, or insurance coverage).

### 2.3 Company website data extraction and validation

For each of the dDPPs with sufficient information for a detailed characterization, we extracted data on several key aspects from their company websites. This website-based data extraction phase was conducted between November 8, 2024, to December 15, 2024. These included the type of platform used (website, app, or both), indicators of program credibility (e.g., number of peer-reviewed publications, CDC recognition status, awards, or certifications), and reported clinical performance outcomes (e.g., weight loss metrics and A1C improvements). Additionally, we examined unique program features such as the use of AI technology, compatibility with devices (e.g., smart body weight scales, continuous glucose monitors), availability of nutrition tracking, and telehealth tools. User experience factors, including satisfaction measures and accessibility, were also evaluated, alongside potential barriers to access, such as limited language options or unclear payment structures.

### 2.4 Data validation

Validation of extracted data was conducted by an independent reviewer (DZ) who was not involved in the original data extraction. Identified discrepancies were resolved through consultation with the senior author (NM), who made the final decision.

### 2.5 Data analysis

Descriptive statistics were employed to summarize the extracted data. Frequencies were calculated to describe characteristics such as program recognition levels, intended audiences, features, and payment options, and were presented as proportions of the total amount of dDPPs. For the subset of dDPPs eligible for the deep-dive analysis, proportions were calculated relative to this group. Inferential statistics were not applicable or necessary, as the review focuses on summarizing dissemination and implementation trends rather than testing hypotheses or making predictions.

## 3 Results

### 3.1 Included DPPs

**Figure 1:**
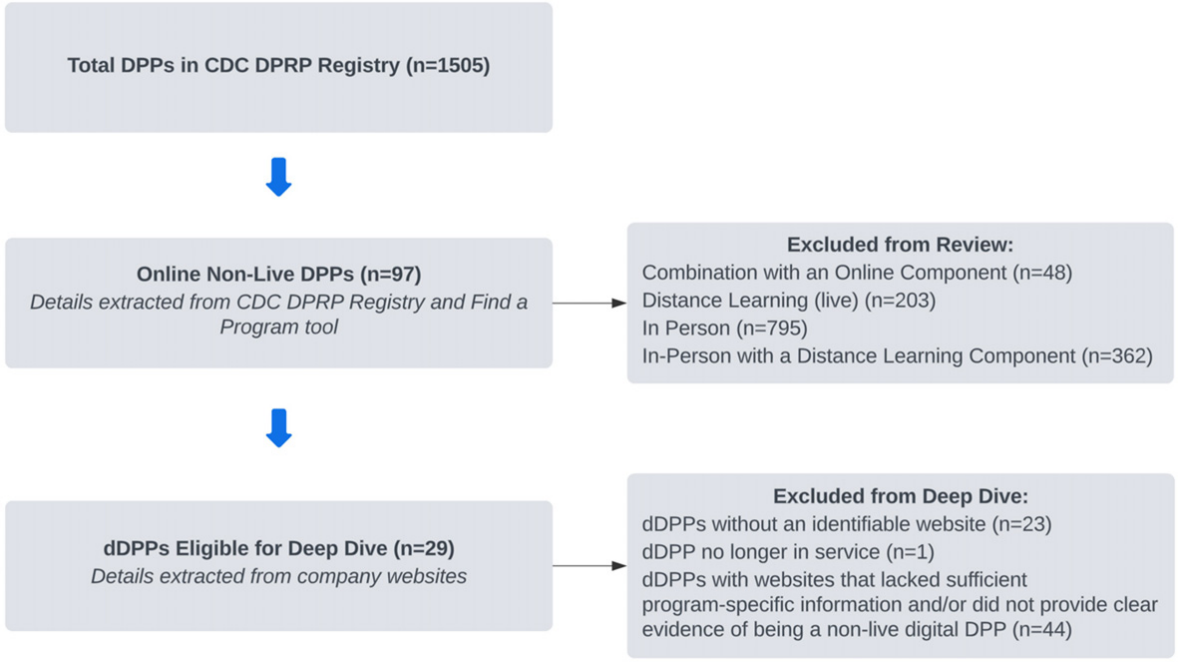
Flow diagram of dDPPs identified from the CDC DPRP Registry. dDPP, digital Diabetes Prevention Program; CDC DPRP, Centers for Disease Control and Prevention Diabetes Prevention Recognition Program.

**Figure 1** illustrates the selection process for identifying dDPPs eligible for inclusion in this review. As of November 8, 2024, the CDC DPRP registry included 1,505 DPPs categorized by their delivery modes: in-person, in-person with a distance learning component, distance learning (live), combination with an online component, and online (non-live) (CDC). Of these, 97 programs were classified as online non-live dDPPs and were eligible for the first component of this review, which aimed to summarize and identify potential gaps within CDC-based resources (Sections 3.2 and 3.3 below). The CDC DPRP registry also included the level of CDC recognition for each DPP, reflecting the program’s progress in meeting the agency’s standards for evidence-based interventions. The definitions and requirements for each level are outlined in **Table 1** (CDC DPRP Standards and Operating Procedures 2024).

**Table 1:** DPP tiering system as defined by the CDC DPRP standards and operating procedures.

Recognition Tier Level	Definition	Key Requirements
Pending	Initial recognition upon application approval.	- Submit a complete application to dprp.cdc.gov - Commit to delivering a yearlong program that follows a CDC-approved curriculum
Preliminary	Awarded for initial success in engaging participants and delivering the program.	- Meet all Pending requirements - Submit data from ≥5 participants who attended ≥8 sessions within a 12–18-month evaluation period
Full	Advanced recognition for demonstrating program quality, retention, and measurable outcomes.	- Meet all Preliminary requirements - Retain ≥30% of participants as “completers” (defined as participants with ≥8 sessions attended and enrolled in ≥9 months) - ≥60% of completers achieve one of: • ≥5% weight loss • ≥4% weight loss + 150 min/week activity (8 sessions) • ≥4% weight loss + attendance at 17 sessions • ≥0.2% A1C reduction - ≥35% of completers qualify via blood test or GDM history
Full Plus	The highest level of recognition, reflecting exceptional participant retention and program quality.	Meet all Full recognition requirements - Retain participants at: • 50% retention at start of month 4 • 40% retention at start of month 7 • 30% retention at start of month 10

CDC, Centers for Disease Control and Prevention; dDPP, digital Diabetes Prevention Program; DPRP, Diabetes Prevention Recognition Program; A1C, glycated hemoglobin; GDM, gestational diabetes mellitus.

### 3.2 Summary of CDC DPRP registry and “Find a Lifestyle Program” locator

**Figure 2:**
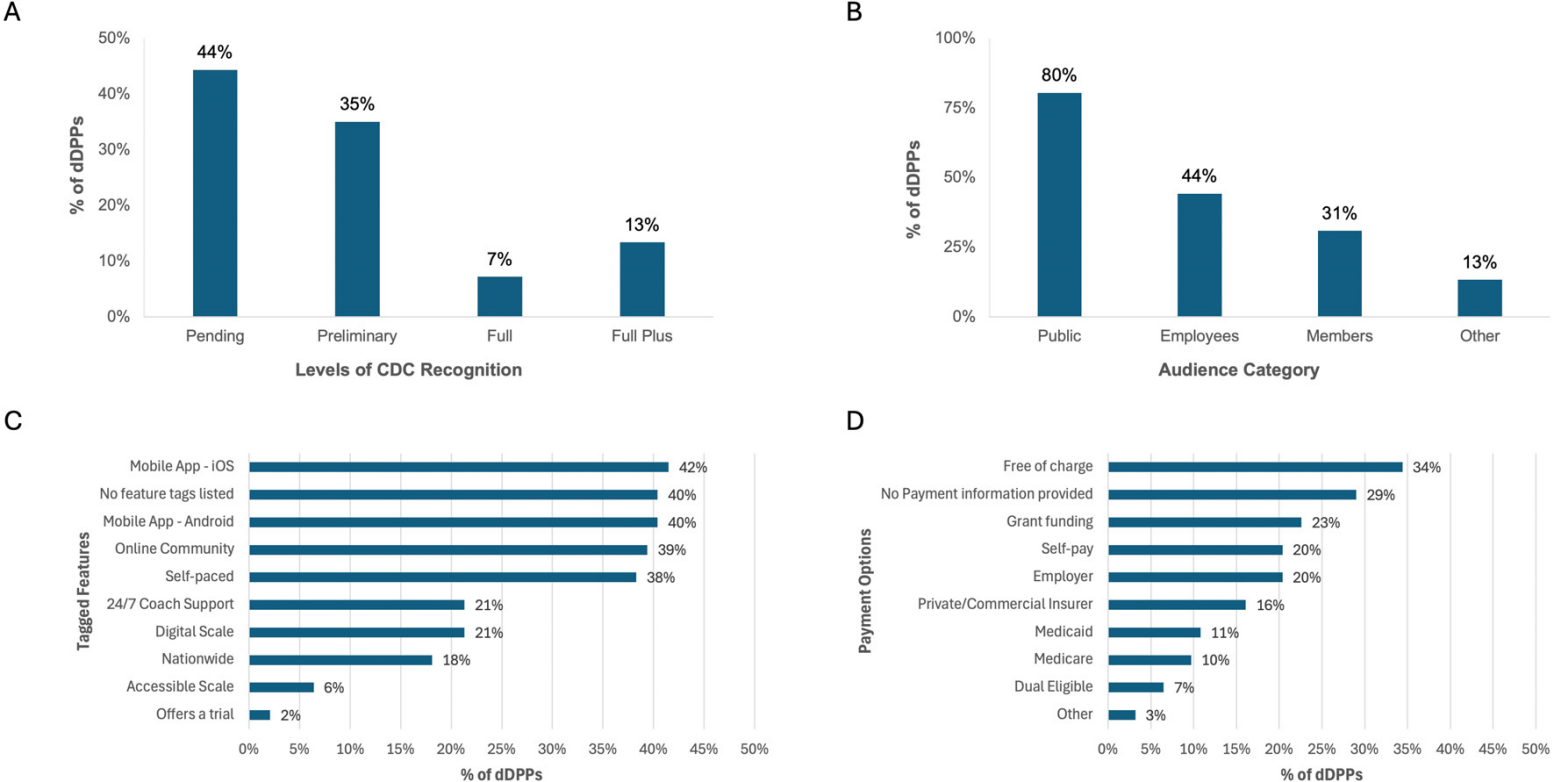
Characteristics of CDC-recognized dDPPs. **(A)** Levels of CDC recognition. **(B)** Intended audience categories. **(C)** Key program features. **(D)** Payment options. Data for **(A, B)** were sourced from the CDC DPRP Registry, while **(C, D)** were derived from the CDC “Find a Program” Locator.

**Figure 2** summarizes the characteristics of the 97 CDC-recognized dDPPs. **Figure 2A** depicts the distribution of CDC recognition levels, with the majority of programs in the early stages of recognition (44% pending, 35% preliminary) and a smaller proportion in the later stages (7% full, 13% full plus). **Figure 2B** highlights the intended audience categories for the 97 dDPPs, with most programs designed for the public (80%), followed by employee-based programs (44%), and member-based programs (31%).

The CDC’s Find a Program Locator offered additional details on the features, languages, and payment options of the dDPPs. However, it is worth noting that three dDPPs listed in the registry were absent from the Find a Program Locator. Nearly half of the programs were delivered through a mobile app (IOS or Android), with over a third featuring an online community and self-paced structures (**Figure 2C**). The majority of programs are offered in English (77%), with a smaller proportion available in Spanish (9%) or English supplemented with Spanish materials (2%). Nearly one-third of the programs were categorized as free of charge, while 20% were classified as utilizing self-pay or employer-based payment options.

### 3.3 Availability and functionality of dDPP websites

**Figure 3:**
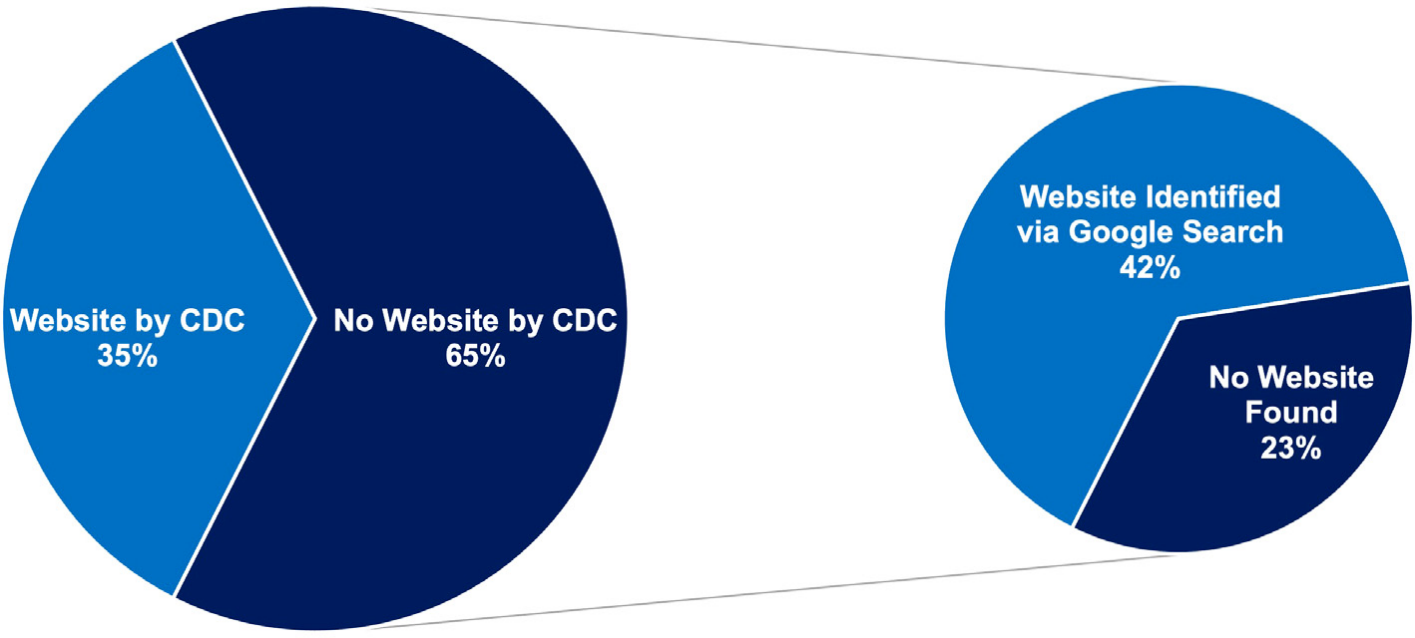
Website availability for CDC-recognized dDPPs. Left pie chart: proportion of dDPPs with websites listed in the CDC DPRP Registry. Right pie chart: additional websites identified through manual searches.

**Figure 3** illustrates the availability of websites for the 97 dDPPs. Within the CDC DPRP Registry, only 35% of the 97 dDPPs had websites listed. Additionally, three of the websites provided by the CDC led to invalid or broken links. However, we were able to identify updated and functional websites for two of these programs after our manual search. For the 63 dDPPs without a website listed in the CDC registry, 41 had associated websites identified through a manual Google search. The websites of the remaining 22 dDPPs could not be identified. A complete list of all 97 dDPPs, including their associated websites as identified by the CDC or through our manual search, is provided in **Supplementary Table 1**.

### 3.4 Detailed characterization of dDPP offerings

Of the 97 identified dDPPs, 29 were eligible for a deep-dive analysis of their associated program structure and features. The remaining 68 were excluded due to either lacking an identifiable website (n=23), no longer being in service (n=1), or having websites that lacked sufficient program-specific information or clear evidence of being a non-live digital DPP (n=44). Data extracted from the 29 dDPPs, including program names, credibility measures, demonstrations of efficacy, and program-specific features, has been compiled into **Supplementary Table 2**.

#### 3.4.1 Demonstration of credibility

**Figure 4:**
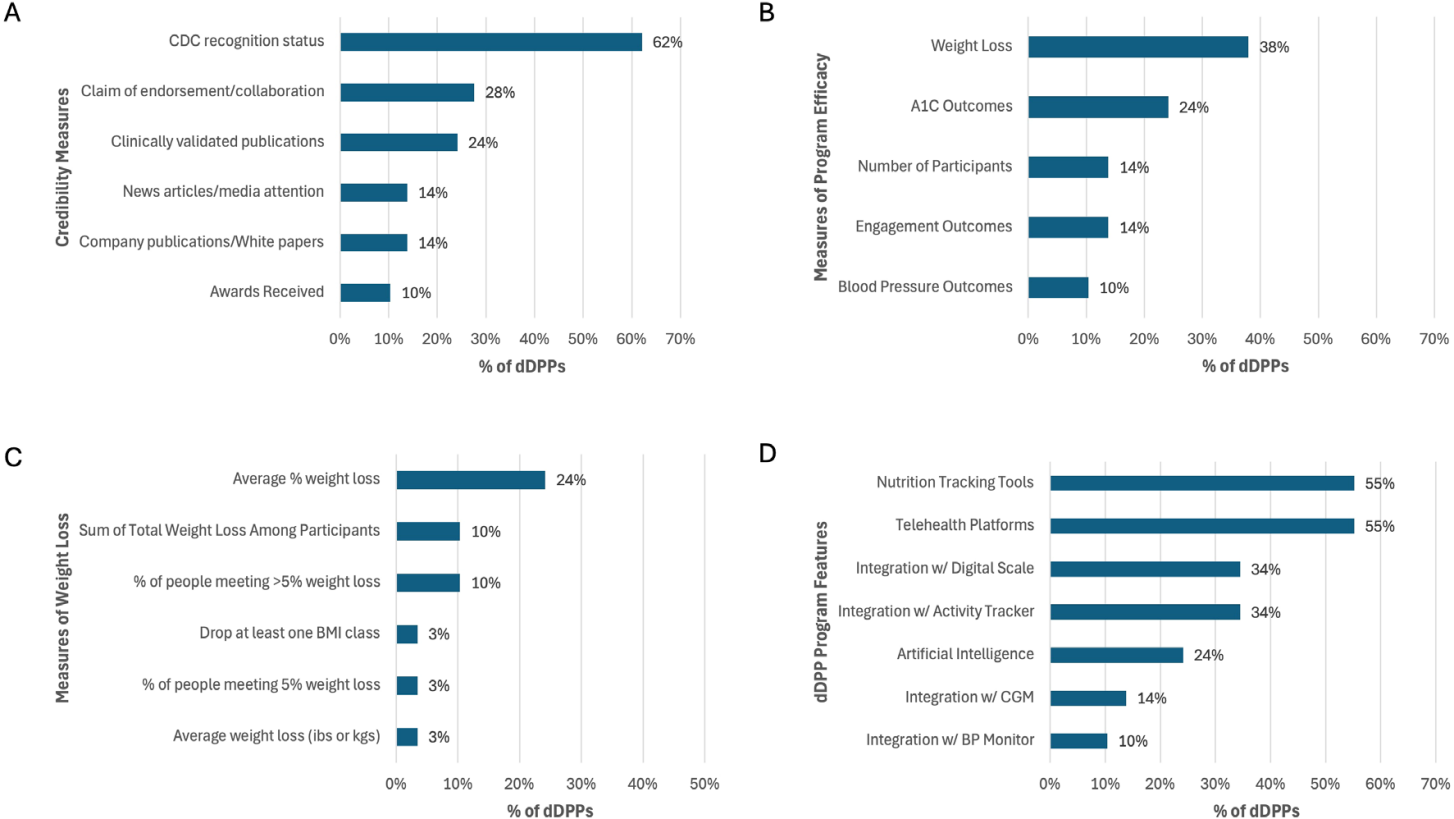
Features of dDPPs with sufficient website data. **(A)** Credibility measures. **(B)** Reported efficacy outcomes. **(C)** Distribution of weight loss metrics. **(D)** Features and tools provided, including AI integration, device compatibility, and telehealth. Data reflect findings from 29 dDPPs. CGM, continuous glucose monitor; BP, blood pressure.

A majority of dDPPs (86%) included information on their websites aimed at establishing credibility with their audiences. This credibility was demonstrated through various means, as shown in **Figure 4A**: 62% highlighted CDC recognition (though most did not specify their level of recognition), 28% claimed endorsements or collaborations with trusted entities such as the American Diabetes Association or public figures, 24% cited peer-reviewed publications, 14% referenced media attention, and 10% showcased awards received. Additionally, 14% of the dDPPs used company-produced whitepapers (i.e., internal reports summarizing program outcomes, methodologies, or claims of effectiveness that are not peer-reviewed).

#### 3.4.2 Clinical performance metrics

Nearly half (48%) of the dDPPs included performance data on their websites, with the selection of program efficacy measures varying widely, as shown in **Figure 4B**. The most commonly reported efficacy metric was weight loss (38%), followed by A1C outcomes (24%) and engagement metrics (14%). Additional measures included the total number of participants to date (14%) and improvements in blood pressure (10%).

**Figure 4C** illustrates the distribution of weight loss metrics reported by dDPPs, with the most frequently reported metric being the average percentage weight loss (24%), followed by the percentage of participants achieving greater than 5% weight loss (10%) and the total weight loss among all participants (10%).

#### 3.4.3 Program-specific features

The dDPPs were delivered through various platforms, with 52% utilizing a mobile app, 35% using both a mobile app and a web page, and 13% relying solely on a web page. **Figure 4D** presents the frequency of various features offered by the dDPPs. AI was used in 24% of programs, with 17% implementing AI-driven health recommendations and 7% utilizing photo-based meal detection for dietary tracking. Additionally, device integration was a common feature. Digital scales were provided by 31% of programs, with 34% offering compatibility for integration. Similarly, 31% of dDPPs provided activity trackers, while 34% supported integration with these devices. A smaller proportion (14%) of programs supported integration with CGMs. Smart blood pressure monitors were provided by 3% of dDPPs, and 10% allowed integration with these devices. Nutrition tracking tools were included in 55% of dDPPs, enabling users to log food intake and nutritional information. Telehealth platforms, facilitating virtual health coaching sessions or consultations, were also offered by 55% of programs.

Many dDPPs represented programs that could address additional health conditions and behaviors beyond prediabetes. The most commonly addressed conditions included obesity and weight management, hypertension, and mental well-being. Other conditions included type 2 diabetes, hyperlipidemia, sleep, coronary heart disease, heart failure, and medication adherence.

#### 3.4.4 User-experience, payment, and access barriers

We found that the majority of dDPP websites included some form of user satisfaction measure. The most commonly reported measure was patient testimonials, which appeared on 69% of dDPPs, providing qualitative insights into user experiences. Satisfaction scores, reported by 24% of dDPPs, offered a quantitative perspective on user satisfaction but were less frequently included.

Additionally, 28% of dDPPs gave information about payment options available. These details typically outlined whether programs were free of charge, self-pay, or covered by insurance or grants, although specifics were not always clearly presented. Furthermore, 14% of dDPPs included information about the languages in which the program was offered.

Notably, 72% of programs provided limited information on their websites, requiring users to submit their name and email address to access additional details.

## 4 Discussion

Our review summarizes the evolving landscape of CDC-recognized digital diabetes prevention programs. Among the 1,505 DPPs within the CDC DPRP registry, only 97 (6%) were non-live digital DPPs, reflecting a nascent but growing approach to diabetes prevention. While this may suggest that digital non-live programs are underrepresented, their inherent scalability offers a unique advantage. Unlike in-person or hybrid programs, a single well-designed digital non-live program has the potential to reach a significantly larger audience, reducing the need for numerous programs if those available are effective. Despite their promise, as highlighted by a recent meta-analysis indicating mobile app dDPPs facilitate modest but meaningful weight loss, much of the evidence underpinning the efficacy of dDPPs is derived from longitudinal, non-randomized studies (15, 16). This reliance on a smaller number of dDPPs and the lack of robust RCT data highlights the urgent need for more rigorous evaluation and validation of these programs to ensure they meet the diverse needs of populations at risk for diabetes.

Our analysis of the CDC DPRP Registry and “Find a Lifestyle Program” navigator revealed an uneven distribution in both program maturity and intended audiences. Most dDPPs were in earlier stages of development, yet to fully meet the CDC’s evidence-based standards. This indicates significant potential for growth and improvement in the dDPP landscape, as many programs are in the pipeline for further development and evaluation. Additionally, the predominance of public-facing programs suggests a focus on broad accessibility but may overlook the potential benefits of tailoring interventions to specific populations with unique needs, such as communities that have been historically marginalized (17). The limited availability of programs in Spanish and those supplementing English with Spanish materials further highlights the need for greater linguistic inclusivity in dDPPs, a barrier seen in other areas of digital health as well (18).

Unlike in-person or remote learning-based programs, which typically follow a standardized cadence and curriculum set by the CDC, digital non-live DPPs have significant freedom to vary in their design and features. This inherent heterogeneity can be advantageous, offering diverse options to meet individual needs, but it also introduces complexity for consumers trying to identify the program that best aligns with their preferences and health goals. This underscores the critical need for centralized databases with accurate, clear, and comprehensive information to enable well-informed decision-making.

Our review identified several potential concerns with the CDC’s information sources for dDPPs. First, three dDPPs listed in the CDC DPRP registry were entirely absent from the “Find a Program” locator, which suggests inconsistencies in how programs are cataloged and displayed. Second, while some dDPPs in the locator included helpful descriptive tags (e.g., “digital scale”), these were inconsistently applied; many programs lacked any descriptor tags, limiting the locator’s utility as a decision-making tool. Third, most CDC-recognized dDPPs did not have a website listed on either the registry or the locator. This issue arose for two main reasons: a lack of comprehensiveness in the CDC’s database (as our manual searches identified 41 missing websites) and the fact that 22 programs were entirely untraceable online. This is particularly concerning, as a functional website is a critical factor in building trust and credibility for any digital health program, with elements such as clear layout, credible authorship, and ease of use playing a significant role in how consumers evaluate program offerings (19). Additionally, online inaccessibility also raises questions about the operational readiness of these programs—if a program lacks a functioning website, it may also lack the infrastructure necessary to effectively deliver digital health services. Finally, many dDPPs with websites provided sparse or incomplete information, and some websites lacked sufficient details to confirm that the programs qualified as non-live digital DPPs. This raises the possibility of miscategorization by the CDC or, alternatively, that the program websites failed to display enough evidence to verify their eligibility. Together, these findings indicate a challenge the CDC is facing in maintaining a comprehensive, accurate, and reliable database of dDPPs and underscore the need for improved oversight, auditing, and standardization to better support consumers and the broader public health mission.

The majority of dDPP websites lacked sufficient information for a deep-dive analysis, possibly because many programs primarily serve employees or members, restricting key details behind firewalls. A subset of dDPPs was eligible for a deep-dive analysis, during which we systematically reviewed program websites to examine the information they provided. We found that the information available on these websites largely fell into one of four categories: program credibility, clinical effectiveness, program-specific features, and user experiences—reflecting diverse ways dDPPs communicate value to potential consumers. However, in the absence of standardized guidelines for website content, we observed significant heterogeneity in the depth and type of information shared, which may affect consumers’ ability to evaluate and compare programs effectively.

Most dDPPs included credibility information, reflecting a shared mission of building potential participants’ trust—a critical element in the successful utilization of digital health technologies dependent on a complex set of factors (20). Within our deep-dive analysis, most companies included information to demonstrate the credibility of their dDPPs, but the approaches varied widely along a spectrum of reliability. At one end, traditionally trustworthy sources such as CDC or ADA endorsements and peer-reviewed scientific publications were used. At the other end, less rigorous methods such as company-produced whitepapers, which lack external validation, were employed. This variability can leave consumers to navigate a mix of highly trustworthy and less reliable claims, which may make it more challenging for individuals to make informed decisions.

As with demonstrations of credibility, measures of program effectiveness included on company websites were remarkably variable. Some programs focused on per-participant metrics, such as average A1C reduction, while others reported aggregate outcomes, such as total weight lost by all participants. This variability in how outcomes are presented may not be helpful to consumers, as it complicates comparisons between programs. Notably, among dDPPs that cited clinical outcomes, the referenced studies were observational or RCTs with control arms that did not represent the standard-of-care landmark DPP (15, 16, 21–25). However, an RCT directly comparing a dDPP to the gold-standard DPP is ongoing, with results anticipated in 2025 (8).

The features offered by today’s dDPPs are innovative, with the integration of devices such as weight scales, blood pressure cuffs, and physical activity monitors indicating a more data-driven approach to diabetes prevention support. However, to our knowledge, there is no research identifying which specific features in a dDPP are more effective than others. Among the most exciting advancements is the use of AI in dDPPs, which is employed in various ways to enhance user experience and provide personalized insights. For instance, Lark utilizes conversational AI to mimic real-time coaching, offering personalized guidance and support through its chatbot platform (26). Heali AI takes a different approach, using photo-based meal detection and optical character recognition to help users accurately log and monitor their dietary habits (27). The growing use of AI in dDPPs stands in contrast to the CDC’s cautious stance on its ability to replace a human Lifestyle Coach, requiring live interaction through email or text during active weeks and explicitly excluding chatbots or AI from fulfilling this role (28).

There were several strengths to this review. First, by leveraging two independent CDC resources—the DPRP Registry and the Find a Program Locator—we obtained complementary datasets that enhanced the breadth of our analysis. Second, the use of data validation procedures, including independent review and resolution of discrepancies by a senior author, minimized errors and improved the reliability of our findings. Third, our structured approach to data extraction, facilitated by REDCap tools, ensured consistency and completeness across diverse data points, from program features to user experience factors. Finally, the inclusion of a deep-dive analysis of company websites allowed for a detailed evaluation of dDPP-specific characteristics, such as AI integration, device compatibility, and accessibility measures, providing novel insights into the current landscape of dDPPs.

Our review has limitations. First, the accuracy of the CDC resources utilized for results is a concern, as discrepancies between the DPRP Registry and the Find a Program Locator, as well as missing or outdated information, may have impacted the accuracy and completeness of the data analyzed. Second, the accuracy of the website data used for the deep-dive analysis is similarly limited, as the findings reflect only the information advertised by companies or presented by the CDC. Without firsthand evaluation of the dDPPs, our analysis is restricted to the consumer-facing details available online, which may not fully represent the actual features, quality, or performance of these programs. As such, the findings provide insights into what a consumer might encounter when selecting a dDPP but do not account for potentially important factors that are not publicly disclosed or marketed. Third, this review does not include other commercially available lifestyle programs (e.g., Levels), which could be used to aid in diabetes prevention but are not recognized by the CDC (29). As a result, our findings are limited to CDC-recognized dDPPs and may not capture innovations or features present in non-recognized programs, which could provide additional insights into the broader landscape of digital diabetes prevention.

Future research and efforts should focus on addressing several key areas to advance the field of dDPPs. First, there is a pressing need for more complete and accurate centralized sources of information, such as the CDC DPRP Registry and the Find a Program Locator, to ensure that consumers have reliable and comprehensive data when selecting a dDPP. This would not go without challenges, as maintaining such a database manually would be difficult given the continuous emergence of new programs and evolving program features. Additionally, there is no clear process for updating or phasing out outdated programs, even if they were once highly regarded. Establishing mechanisms for continuous monitoring and program comparability will be important to ensure consumers can identify the most effective options. A community-driven approach, involving real-world data and feedback from patients and other stakeholders, could help keep evaluations relevant over time. Second, dDPP websites should strive to become more functional and informative, offering clear, accessible, and detailed descriptions of program features, outcomes, and trustworthy demonstrations of credibility to enhance transparency and usability for potential participants. Third, further research is needed to evaluate the effectiveness of dDPPs, with particular attention to identifying which program features are most successful in specific populations. Studies exploring the impact of technologies such as AI, device integration, and telehealth on clinical outcomes will be essential for guiding future program development and improving the personalization of diabetes prevention strategies.

## 5 Conclusion

This study provides a consumer-oriented review of CDC-recognized digital diabetes prevention programs, highlighting their innovative features, variable designs and structures, and gaps in online accessibility and presentation. Our findings emphasize the need for improved centralized surveillance, as discrepancies and missing information in the CDC’s databases hinder consumer decision-making and program transparency. dDPPs have the potential to deliver personalized and scalable diabetes prevention support, especially in the context of AI integration and other advanced technologies. It should be noted, however, that the lack of robust evidence on the effectiveness of these features, as well as dDPPs overall, underscores a significant gap in the existing literature.

Future efforts should prioritize creating more complete and accurate centralized sources of information, enhancing the functionality and transparency of program websites, and conducting rigorous research to evaluate the effectiveness of dDPPs. Addressing these gaps will be essential for optimizing the development, reach, and impact of dDPPs.
