# Supplementary material for: Consumer-oriented review of digital diabetes prevention programs: insights from the CDC’s diabetes prevention recognition program
Source: Front Clin Diabetes Healthc. 2025 Apr 16;6:1562108. doi: 10.3389/fcdhc.2025.1562108 (PMC12040819; doi:10.3389/fcdhc.2025.1562108)
Supplement: Supplementary file 3 [file DataSheet1.pdf]

## Table of Contents


1. **CDC Registry Data Collection Instrument**
   *(Page 2)*
2. **CDC "Find a Lifestyle Program" Data Collection Instrument**
   *(Page 3)*
3. **dDPP Website Data Collection Instrument**
   *(Page 5)*

# CDC Registry Data

| Field | Response |
|---|---|
| Record ID | __________________________________ |
| DPP Name | __________________________________ |
| Address Line 1 | __________________________________ |
| Address Line 2 | __________________________________ |
| City | __________________________________ |
| State | __________________________________ |
| Zip | __________________________________ |
| Phone | __________________________________ |
| Email | __________________________________ |
| Website listed | ◯ Yes<br>◯ No |
| Web Address | __________________________________ |
| Recognition Status | ◯ Pending<br>◯ Preliminary<br>◯ Full<br>◯ Full Plus |
| Class Open to | ☐ Public<br>☐ Employees<br>☐ Members<br>☐ Other |

# Find a Program Data

Record ID ______________________

Abstract information about each Digital DPP, also referred to as online (non-live), programs from this page: https://www.cdc.gov/diabetes-prevention/lifestyle-change-program/find-a-program.html

Name of data abstractor
- ◯ Benjamin Lalani
- ◯ Jalene Shim
- ◯ Vidhu Vadini
- ◯ Yllka Valdez
- ◯ Nestoras Mathioudakis

The DPP name as listed on the CDC registry is: [dpp_name].

The state listed is: [state]

Could the online program be identified on the CDC's Find a Program webpage?
- ◯ Yes
- ◯ No

Name of the program as listed on "Find a Lifestyle Change Program" ______________________

Is Link to "Visit Website" Provided?
- ◯ Yes
- ◯ No

Concordance between CDC Registry and Find a Program (website listed) ______________________
(0 = Neither; 1= CDC registry only; 2= Find a Program Only; 3 = Both)

List the url of the website link provided from the Find a Program Page ______________________

Concordance between CDC Registry and Find a Program (URL) ______________________
(1= match; 0= do not match)

Is the website link valid?
- ◯ Yes
- ◯ No

What happens when you click the link? ______________________

Which of the following Features tags are listed on Find A Program?
- ☐ Nationwide
- ☐ Digital Scale
- ☐ Accessible Scale
- ☐ 24/7 Coach Support
- ☐ Mobile App- Android
- ☐ Mobile App- iOS
- ☐ Offers a trial
- ☐ Online Community
- ☐ Self-paced
- ☐ No feature tags listed

Language Options

- ☐ English
- ☐ Spanish
- ☐ English but supplemented with Spanish materials
- ☐ Chinese
- ☐ English but supplemented with Chinese materials
- ☐ American Indian or Alaska Native language
- ☐ Native Hawaiian or Other Pacific Islander language or dialect
- ☐ English but supplemented with Native Hawaiian or Other Pacific Islander language or dialect material
- ☐ English but supplemented with American Indian or Alaska Native Language materials
- ☐ Other
- ☐ No language information provided (no feature tag)

Payment Options

- ☐ Free of charge
- ☐ Private Insurer
- ☐ Employer
- ☐ Medicare
- ☐ Medicaid
- ☐ Self-pay
- ☐ Dual Eligible
- ☐ Grant funding
- ☐ Private/Commercial Insurer
- ☐ Other
- ☐ Government/Military
- ☐ Venture Capital
- ☐ No Payment information provided

CDC-Recognized Organization Associated with this Site

________________________________

(Cut and paste name, address, phone number)

## Validation

Name of data validator

- ◯ Benjamin Lalani
- ◯ Jalene Shim
- ◯ Vidhu Vadini
- ◯ Yllka Valdez
- ◯ Nestoras Mathioudakis
- ◯ Daniel Zade

Date of data validation

________________________________

Validation Issues/Comments

________________________________

# Company Website Data - Organized

| | |
|---|---|
| Record ID | ______________________________ |
| Name of data abstractor | ◯ Benjamin Lalani<br>◯ Jalene Shim<br>◯ Vidhu Vadini<br>◯ Yllka Valdez<br>◯ Nestoras Mathioudakis |
| Can you find a website associated with this DPP? | ◯ Yes<br>◯ No<br>(either from CDC registry, Find a Program, or Google search) |
| URL on CDC Registry: [web_address]<br>URL on Find a Program: [list_url_find_a_program] | |
| List url of company website (as specific as possible for DPP) | ______________________________<br>(If the link provided on the CDC Registry or Find A Program is not valid but you are able to find the correct URL through a Google search, list here.) |
| Does company website provide specific and detailed information about the DPP? | ◯ Yes<br>◯ No<br>(Beyond generic description of what the National DPP is -- i.e. specific aspects of the diabetes prevention program need to be mentioned.) |
| Does it seem like the digital (online) DPP may have been misclassified (i.e. distance learning video conference or in-person sessions)? | ◯ Yes<br>◯ No |
| Is there evidence the DPP is out-of-service or not currently available? | ◯ Yes<br>◯ No |
| Type of Digital DPP | ◯ Proprietary (developed by company)<br>◯ External DPP (e.g. HALT) offered<br>◯ Unclear |
| URL of external DPP | ______________________________ |

## Program Credibility

| | |
|---|---|
| Credibility-Related Information Listed on Company Website | ☐ Clinically validated publications<br>☐ Company publications/ White papers<br>☐ CDC recognition status (i.e. Full, Full Plus, etc.)<br>☐ Claim of endorsement/collaboration (e.g. ADA, celebrity)<br>☐ Awards Received<br>☐ News articles/ media attention<br>☐ Other |

| | |
|---|---|
| Number of clinically validated (i.e. peer reviewed publications specifically on the DPP) | ________________________ |
| Are links to the actual publications provided? | ◯ Yes<br>◯ No |
| Number of company publications/whitepapers (specific to Diabetes) | ________________________ |
| Are links to the company white papers/publications actually provided? | ◯ Yes<br>◯ No<br>(This is non peer-reviewed papers. Mark no if individual must provide their contact information in order to get the paper.) |
| What CDC recognition status is listed on company website | ◯ Pending<br>◯ Preliminary<br>◯ Full<br>◯ Full Plus<br>◯ Not specified |
| List professional groups/societies/companies/celebrities that the company claims to have endorsed or collaborated with their product | ________________________<br>(separate each entity with comma. For example, American Heart Association, American Diabetes Association) |
| Number of awards received | ________________________ |
| List names of awards received | ________________________<br>(name award) |
| News Articles / Media Attention | ________________________<br>(separate each article title by a comma) |
| Other Claim of Program Credibility | ________________________ |

## Clinical Performance/Outcomes

| | |
|---|---|
| Information listed on Company Website | ☐ Weight loss<br>☐ Physical activity outcomes<br>☐ A1C outcomes<br>☐ Engagement outcomes (e.g., retention)<br>☐ Number of members/participants<br>☐ Other |
| What type of weight loss outcome is reported? | ☐ Average weight loss (lbs or kg)<br>☐ Average % weight loss<br>☐ % of people meeting 5% weight loss<br>☐ % of people meeting >5% weight loss<br>☐ Other |

| | |
|---|---|
| Average Weight Loss (ibs or kgs) | __________________ |
| Average % Weight Loss | __________________ |
| % of people meeting 5% weight loss | __________________ |
| % of people meeting >5% weight loss | __________________ |
| Other Weight Loss Outcome | __________________ |
| What type of physical activity outcome is reported? | ☐ Average minutes / week of physical activity<br>☐ % of participants meeting 150 minutes of activity / week<br>☐ Average increase in minutes/week of physical activity<br>☐ Other |
| Average minutes / week of physical activity | __________________ |
| % of participants meeting 150 minutes of activity / week | __________________ |
| Average increase in minutes/week of physical activity | __________________ |
| Other physical activity outcome reported | __________________ |
| What type of A1C outcomes are reported? | ☐ Average A1C<br>☐ Average change in A1C<br>☐ % of participants meeting A1C decrease of 0.2 or greater<br>☐ % of participants with normal A1C after completing program<br>☐ Other |
| Average A1C | __________________ |
| % of participants meeting A1C decrease of 0.2 or greater | __________________ |
| % of participants with normal A1C after completing program | __________________ |
| Average change in A1C | __________________ |
| Other A1C Outcome | __________________ |

| | |
|---|---|
| What type of engagement outcomes are reported? | ☐ Program Completion Rate<br>☐ Lesson Completion<br>☐ Daily Logins<br>☐ Weekly Active Users<br>☐ Food Logging Frequency<br>☐ Physical Activity Tracking Frequency<br>☐ Weight Tracking Frequency<br>☐ Time Spent on Platform<br>☐ Feature Adoption Rate (% of participants using specific features)<br>☐ Responsiveness to prompts/reminders<br>☐ Retention Rate (% of participants who remain active over a specified period)<br>☐ Other |
| Program Completion Rate | __________________________ |
| Lesson Completion | __________________________ |
| Daily Logins | __________________________ |
| Weekly Active Users | __________________________ |
| Food Logging Frequency | __________________________ |
| Physical Activity Tracking Frequency | __________________________ |
| Weight Tracking Frequency | __________________________ |
| Time Spent on Platform | __________________________ |
| Feature Adoption Rate (% of participants using specific features) | __________________________ |
| Responsiveness to prompts/reminders | __________________________ |
| Retention Rate (% of participants who remain active over a specified period) | __________________________ |
| Other Engagement Outcome | __________________________ |
| List number of members/participants company claims to have used their product | __________________________ |
| Other Clinical Performance Measures | __________________________ |

## Program-Specific Features

Platform
- ◯ Mobile app
- ◯ Web page
- ◯ Both

Program Specific Features
- ☐ Technology offered (e.g., digital scale, Fitbit)
- ☐ Technology integration (e.g., Apple Watch, Google Fit)
- ☐ AI technology (e.g., chatbot, photo-based meal detection)
- ☐ Health information dashboard on website/app
- ☐ Automated reminders (e.g., medication timing, meal timing, SMS / app notifications)
- ☐ Human coaching option
- ☐ Self-paced program
- ☐ Informational/demo videos about program
- ☐ Educational video libraries
- ☐ Expected duration of program
- ☐ Food subscriptions / meal delivery / etc.

Expected Duration of Program

________________________________
(enter in months)

AI technology
- ☐ AI-driven health recommendations
- ☐ Photo-based meal detection
- ☐ Other

(enter in months)

AI technology - Other

________________________________
(enter in months)

Which of the following tags can be inferred from the company website?
- ☐ Nationwide
- ☐ Digital Scale
- ☐ Accessible Scale
- ☐ 24/7 Coach Support
- ☐ Mobile App- Android
- ☐ Mobile App- iOS
- ☐ Mobile App- not specified
- ☐ Offers a trial
- ☐ Online Community
- ☐ Self-paced

## Technologies Provided or Capable of Integrating

| | Offered (physical device or in-app feature) | Integration Possible (with external device or external app) |
|---|---|---|
| Digital scales (monitor weight/transmit data automatically) | ☐ | ☐ |
| Activity trackers (e.g. Fitbit, Garmin, Apple Watch) | ☐ | ☐ |

| | | |
|---|---|---|
| CGMs (e.g. Dexcom, Libre) | ☐ | ☐ |
| Smart Blood Pressure Monitors | ☐ | ☐ |
| Mobile apps - customized program apps for tracking goals, diet, and exercise | ☐ | ☐ |
| Wearable heart rate monitors (tracking cardiovascular metrics) | ☐ | ☐ |
| Sleep trackers | ☐ | ☐ |
| Nutrition tracking tools (apps/devices to log food intake and nutritional info) | ☐ | ☐ |
| Telehealth platforms (virtual health coaching sessions/consultations) | ☐ | ☐ |
| Digital food scales (portion control, calorie tracking) | ☐ | ☐ |
| Glucose Meters for periodic blood glucose checks | ☐ | ☐ |
| Other technology | ☐ | ☐ |

Describe other technology

__________________________________

Does the digital platform extend to health conditions or behaviors besides prediabetes (e.g. sleep, mental health, type 2 diabetes, etc.?

○ Yes
○ No

Which other health conditions or behaviors does the digital platform extend to besides prediabetes?

☐ Obesity/weight management
☐ Hypertension
☐ Type 2 diabetes
☐ Heart failure
☐ Medication adherence
☐ Mental wellbeing
☐ Sleep
☐ Coronary heart disease
☐ Hyperlipidemia
☐ Other

Other health conditions not listed above

__________________________________

## User Experience and Support

Information listed on Company Website

☐ Satisfaction scores (e.g., App Store rating, Net Promoter Score)
☐ Patient testimonials
☐ FAQ page

What type of satisfaction scores are reported?

☐ App Store rating
☐ Net Promoter Score (NPS)
☐ Customer Satisfaction Score
☐ Other

Net Promoter Score

________________________

Describe other satisfaction measure

________________________

## Eligibility, Payment, Access Barriers

Information listed on Company Website

- ☐ Eligibility (payment options)
- ☐ Provide contact information (Name/Email) to learn more about program
- ☐ Languages offered
- ☐ Other

Other Eligibility / Access-related Information

______________________________

Language Options

- ☐ English
- ☐ Spanish
- ☐ English but supplemented with Spanish materials
- ☐ Chinese
- ☐ English but supplemented with Chinese materials
- ☐ American Indian or Alaska Native language
- ☐ Native Hawaiian or Other Pacific Islander language or dialect
- ☐ English but supplemented with Native Hawaiian or Other Pacific Islander language or dialect material
- ☐ English but supplemented with American Indian or Alaska Native Language materials
- ☐ Other
- ☐ No language information provided (no feature tag)

Other Language Offered

________________________

## Validation

Name of data validator

- ◯ Benjamin Lalani
- ◯ Jalene Shim
- ◯ Vidhu Vadini
- ◯ Yllka Valdez
- ◯ Nestoras Mathioudakis
- ◯ Daniel Zade

Date of data validation

________________________

Issues/Comments identified during data validation

______________________________
